# Nurses and Doctors Heroes? A Risky Myth of the COVID19 Era

**DOI:** 10.3390/nursrep10020006

**Published:** 2020-09-28

**Authors:** Carlo V. Bellieni

**Affiliations:** Ethics Committee of the Tuscany Region and Pediatric Intensive Care Unit, University Hospital of Siena, 53100 Siena, Italy; carlo.bellieni@ao-siena.toscana.it

**Keywords:** COVID-19, pandemic, medical humanities

## Abstract

Recent newspapers reports have named health professionals as “heroes”. This is surprising, because in the last few decades, doctors and nurses have been taken into account by mass media only to describe cases of misconduct or of violence. This change was due to the coronavirus pandemic scenario that has produced fear in the population and the need for an alleged “savior”. This need for health professionals seen as heroes is also disclosed by the fact that even politicians have abdicated to their role in favor of the healthcare “experts” to whom important decisions on social life during this pandemic have been delegated, even those decisions that fall outside of the specific health field. This commentary is a claim to framing the job of caregivers in its correct role, neither angel nor devil, but allied to the suffering person, that the image of “heroes” risks to overshadow.

Until a few months ago, the medical chronicles were full of reports showing increasing episodes of violence against doctors and nurses [1]. The newspapers used to report clinical news mainly about cases of misconduct or medical errors. Suddenly, in the shadow of the nefarious coronavirus, one reads only of healthcare heroes: On 24 March, an article appeared on the CNN site titled “The real modern heroes are the health workers”, and on 24 April, on this track the BBC asked: “Will coronavirus change how we define heroes?” with reference to the efforts of COVID-19 caregivers. “From rags to riches”, we would say; but careful: As it is commonly said, “all that glitters ain’t gold” because there is a high risk that all this sanctification may end soon and lead again to a scenario where health professionals are unsatisfactory again. The aim of this commentary is to point out this paradox and to warn against the illusion that this really represents a correct vision of caregivers’ role. 

Across the centuries, physicians, due to increasing knowledge and technique, have become something like “demi-gods” for the public. This perspective has been questioned in more recent decades due to a certain emancipation of the public and the criticism toward scientists, including physicians. For nursing, it is not quite the same as with physicians, because nurses have emerged as a role more recently. In antiquity and in later centuries, persons who acted as nurses or midwives were either revered or blamed as witches. In more recent centuries, particularly with the growing influence of Florence Nightingale, nursing has moved into a more scientific sphere, obtaining a higher regard. However, in recent decades, nursing—for example, in Europe—has been struggling for recognition and has itself failed to demonstrate the potential that nurses bring to the table [2]; the public has lost interest and trust in nursing to some extent and the reasons for this decline will be explained later in this text. 

In more recent years, healthcare professionals have often been in a negative light, and the reason seems evident: The assimilation of nurses and doctors to any other job by measuring costs, effects, and users’ satisfaction to appreciate their efforts. The caregiver–patient relationship has been reduced to a contract, and in the absence of the “customer’s satisfaction” or of the sometimes-unrealistic fulfillment of the contract clauses, many pressed legal charges. The caregiver–patient relationship has become a contractual relationship [3], as if the concepts of mutual trust and respect were an insignificant corollary: 3000 years of healthcare history based on the Hippocratic concept have been put in a closet and locked up. However, if the doctor–patient and the nurse–patient relationship has turned into a contract between an operator and a client, and if the hospitals have become “companies”, mistrust skyrockets, then dissatisfaction, and eventually intolerance follow. Ivan Illich explained this well in his book Medical Nemesis [4]: Having ceased curing the person and started curing diseases, the role of doctor and nurses turns against itself in a sort of nemesis, a consequence of a society who sees everything as laws to be followed, contracts, and operating instructions, and as service of a person who got sick and wants—Illich wrote—«to find in the eyes of the doctor a reflection of his own anguish and some recognition of uniqueness of his own suffering». 

In the last few weeks, this scenario has been subverted, exceeding on the other side of the coin. With the deadly threat of the coronavirus, people have felt alone, afraid, and have seen that nurses and doctors are not only “health workers”, but they have, strangely enough, gone too far in this reevaluation: Now, the collective imagination identifies them as heroes. There is too much idealization in this: Doctors and nurses are not to be identified with “misconduct”, but they are neither supermen and superwomen. People seem to adore them, and even politicians tend to abdicate to their decisional power in favor of doctors: “scientific experts” are, these days, asked to supply them strong certainties [5]. This discloses an initial flaw: Experts cannot give strong and definitive certainties, at least in the ultimate mode that they are asked to give, because experts respond to what they are asked for; so, if the only request is to inform politicians regarding when the quarantine shall end, they cannot answer because this answer requires considering not only health issues, but also economic and social aspects of the COVID pandemic [6,7], and also because the epidemiologist can respond about epidemiology, and the virologist or the intensivist only for their respective fields.

Delegating political issues to technicians, and in this case to healthcare experts, reflects a theme that has been widely debated in the philosophic arena. Hannah Arendt in 1958 wrote the book The Human Condition, in which she explained that for the ancient philosophers, human activity was divided into three levels: Simple manual work, and technical and political work, both a function of a higher level activity: Contemplative activity. However, the idea of contemplation has been overshadowed, and politics has lost importance in favor of technical activity. Politics has shifted “from the old questions of ‘what’ and ‘why’ to the new question of ‘how’“ with “the belief that every problem can be solved and every human motivation reduced to the principle of utility; we consider everything that is given as raw material and we see nature as an immense fabric from which we can cut out what we want“ [8]. Furthermore, today, political, moral, and even religious activity seem to be subordinated to the technical data, which cannot however give social or normative granitic certainties; politics is no longer a function of elevated principles or high human functions (philosophy or ethics). So we live in expectation of a definitive and far-sighted response that would arrive from a place from where it cannot come: Technique.

This is the risk of a disembodied and disenchanted Technique, aimed at the useful and not at beauty, as Enzo Tiezzi, a chemist and politician, would have liked [9]. The technician (biologist, architect, doctor, teacher, nurse) is no longer educated to look up, but simply to respond to his job/function. What is worrisome is not the finalism of technology, but the step backward that human conscience, culture, and politics takes, transforming ethics and virtues following a slavish follow-up of finalistic technical protocols. Protocols are not wrong, but they cannot show a wide and futuristic project. Gunther Anders explained that western man lives by an envy towards technology, of which he would like to be a mechanism among others, to lose unpredictability and fantasy for the benefit of a gray wellbeing and routine [10]. Additionally, Umberto Galimberti says that “we continue to think that we have technology as a tool at our disposal. It is not true; it is absolutely not true. Technique has now become the subject of the world and men have turned into apparatuses of this technique. If the technique becomes the universal canon to achieve any purpose, it is no longer an instrument but the first and pervasive purpose of existence” [11]. It is not a mere coincidence that people look for heroes in a disorienting era, and in the midst of a disorienting pandemic. Doctors are being glorified after having been under attack; but let us quote Bertoldt Brecht: “Unfortunate the country that needs heroes” [12]. Brecht did not want to mock heroes, but he pitied those who need them, because this happens when people live in silent fear a solitary, grey life, as well as when those who are responsible for politics and moral issues have abdicated to their role in favor of those who manage technology.

Today, doctors and nurses are certainly in the trenches, in contact with a bad enemy: COVID19. Many give much more than what is required by the protocols, many are encouraged to do that, and we see how much interregional and international solidarity arose in recent days [13]. However, the idealization of the caregivers as heroes is certainly unexpected and undue, as if people were looking for something decidedly more than a strong ally in the doctor: A sacred granitic security.

This heroic glorification risks to be a soap bubble: If the system does not change, this scenario of “Hallelujah” towards nurses and doctors is to be short-lived. Most doctors and nurses are unsatisfied and tired of uselessly asking for a better health system. This glorification will be a bubble if those who want to be gratified by their job will go on only being considered “employees”, engaged with their patients not by trust but by a contract. Doctors and nurses need a health system where the caregiver–patient relationship is not all based on rapid and extorted information, where caregivers do not hide behind cold pages of illegible consents to be signed [14], where health workers have continuous motivations [15], and in which humanity is not overshadowed by bureaucracy, a plethora of useless clinical tests, and protocols [16]; where nurses belong to a world “that focuses on the human-universe-health process articulated in the nursing framework and theories” [2]. Let us take advantage of this transitory scenario, to reaffirm what really nurses and doctors are, what they want, and what people can realistically expect from them.

The role of the politics should no more be to transfer to the experts their own role of decision makers and coordinators of public life, because health professionals are only one group of the likely experts who can provide advice to the decision-makers. At the same time, caregivers should receive more motivation because they are not heroes, or saints; they neither are mere employees, but the images of “heroes” risk to overshadow their real role.

## References

[1] Thomas J.A., Thomas J.J., Paul A.B., Acharya S., Shukla S., Rasheed A., Pratapa S.K. (2019). Medical vandalism: Awareness and opinions; beyond the clinician’s window. J. Family Med. Prim. Care.

[2] Barrett E.A.M. (2017). Again, what is nursing science?. Nurs. Sci. Q..

[3] Bellieni C.V. (2018). Consumerism: A threat to health?. J. R. Soc. Med..

[4] Illich I., Boyars M. (1976). Medical Nemesis.

[5] This Is Who Americans Trust About Coronavirus Information. https://www.washingtonpost.com/politics/2020/03/20/were-all-anxious-about-pandemic-who-do-americans-want-hear/.

[6] Van Gelder N., Peterman A., Potts A., O’Donnell M., Thompson K., Shah N., Oertelt-Prigione S. (2020). COVID-19: Reducing the risk of infection might increase the risk of intimate partner violence. EClinicalMedicine.

[7] Tummers J., Catal C., Tobi H., Tekinerdogan B., Leusink G. (2020). Coronaviruses and people with intellectual disability: An exploratory data analysis. J. Intellect. Disabil. Res..

[8] Arendt H. (1958). The Human Condition.

[9] Tiezzi E. (1998). La Bellezza e la Scienza.

[10] Anders G. (1956). The Outdatedness of Human Beings 1. On the Soul in the Era of the Second Industrial Revolution.

[11] Galimberti U. (2006). Se la Tecnica Distrugge la Natura.

[12] Brecht B., Willett J., Manheim R., Willett J. (1980). Life of Galileo. Collected Plays: Five.

[13] Smith G.D., Ng F., Li W.H.C. (2020). COVID-19: Emerging compassion, courage and resilience in the face of misinformation and adversity. J. Clin. Nurs..

[14] Bellieni C.V., Coradeschi C., Curcio M.R., Grande E., Buonocore G. (2018). Consents or waivers of responsibility? Parents’ information in NICU. Minerva Pediatr..

[15] Mathew R. (2020). Rammya Mathew: Why are doctors so unhappy?. BMJ.

[16] Schwartz B. (2015). Why We Work.

